# Improving Understanding of Screening Questions for Social Risk and Social Need Among Emergency Department Patients

**DOI:** 10.5811/westjem.2020.5.46536

**Published:** 2020-08-20

**Authors:** Gia Ciccolo, Alexa Curt, Carlos A. Camargo, Margaret Samuels-Kalow

**Affiliations:** * Massachusetts General Hospital, Department of Emergency Medicine, Boston, Massachusetts; † Harvard Medical School, Massachusetts General Hospital, Department of Emergency Medicine, Boston, Massachusetts

## Abstract

**Introduction:**

With recent healthcare policy changes, including the creation of accountable care organizations, screening for social risks such as food and housing insecurity has become increasingly common in the healthcare system. However, the wide variety of different tools used for screening makes it challenging to compare across systems. In addition, the majority of tools used to measure social risks have only been tested in primary care settings and may not be optimal for emergency department (ED) use. Therefore, the goal of this study was to create a brief social screening tool for use in EDs.

**Methods:**

We developed an initial tool using publicly available questions corresponding to the five core categories of the Centers for Medicare & Medicaid Services’ Accountable Health Communities Screening Tool. Iterative cycles of cognitive interviews with purposively sampled participants were performed using a hybrid model of think-aloud and verbal probing to understand/experience answering questions and potential comprehension challenges. After thematic saturation was reached in one cycle, the tool was changed per participant input; cycles were completed until thematic saturation was reached overall.

**Results:**

A total of 16 participants (six patient guardians and 10 patients) completed cognitive interviews throughout three cycles. Participant feedback included suggestions for further clarification and simplification of survey questions for improved comprehension. The survey was thus reduced and simplified from 16 questions concerning five domains to 10 questions concerning four domains.

**Conclusion:**

We used an iterative cognitive interviewing process to develop a social screening tool for use in EDs. This process demonstrates the importance of patient input to refine questionnaires, and provides a brief screening tool for ED use.

## INTRODUCTION

With recent policy changes, including the movement toward accountable care organizations as health delivery systems, there has been an increasing priority placed on both screening for social risk factors,^1^–^3^ (defined as the “adverse social conditions that are associated with poor health”)^4^ and assessing social needs, or the patient’s prioritization of social interventions.^4^ Although emergency department (ED) patient populations have a high prevalence of social risk,^5^ optimal strategies for identifying these factors within the busy and time-limited setting of the ED have yet to be described.

Currently, a major barrier to identifying and addressing social risk and social need in EDs is the wide variety of different tools used across studies^5^ and the lack of a “criterion standard” assessment. As stated in a systematic review of social needs in the ED, “a concise yet comprehensive material needs [social risk and social need] screening tool has not yet been created and validated for ED patients.”^5^ Efforts to standardize screening questions^3^ have been limited by the copyright restrictions on recommended questions and the total length of the survey, which limits the applicability of most tools in the ED. To date, studies using shorter tools have focused on screening for an individual social risk (eg, food insecurity) rather than assessing the multiple social risks that may affect patients’ health.^5^–^7^ Few tools assess both social risks and social needs in one assessment. The lack of consistency in ascertainment techniques and screening tools presents an obstacle to researchers, policy makers, and health systems to design interventions to address social risk and social need in the ED patient population.

In developing a social screening tool, it is of critical importance to ensure the screening questions are easy to understand and interpreted in a consistent manner.^8^ Cognitive interviewing has been proposed as a method for improving the validity of response processes, by allowing the researcher to understand how participants interpret questions.^7^ The hybrid cognitive interview methodology involves two parts. The first is “think aloud” in which the intention of the interviewer is to guide the participant in providing verbal insight into his/her thought process and understanding while walking through the survey. The second is “verbal probing” in which the participant responds to specific probes concerning understanding of certain areas of the survey. Survey changes informed by this process ensure that the respondent is interpreting and responding to questions as intended in the survey.^8^ Survey changes based on information from cognitive interviewing data, such as those in this study, are used to clarify the intention of the question to the reader, improve survey comprehension,^4^ and have been used to optimize other self-report assessment tools.^5^,^9^ Modifying a screening tool using this technique can thus increase the ability of the tool to assess risks and needs consistently.

The goal of this study was to develop and optimize a social risk and social need screening tool for ED patients that would be both brief and understandable to patients in order to connect them to potential interventions.

## METHODS

We conducted a cognitive interview study with patients and parents of patients in the ED. The initial 16-question survey was developed in both English and Spanish, through a systematic review of existing social risk and social need screening tools using web-based searches and PubMed. Questions were included if they addressed one of the five core domains of the Accountable Health Communities screening tool: 1) food insecurity; 2) housing instability; 3) transportation needs; 4) utility needs; and 5) interpersonal safety.^3^ This tool was available in the public domain, without copyright restrictions.

A cognitive interview guide of open-ended questions (Online Supplement Table 1) was developed by the study team, piloted and refined. Edits were made to best capture patient understanding and feedback concerning the proposed questions. The guide was developed in English and Spanish, with interviews in the patient’s choice of language. Iterative cycles of cognitive interviews were performed and recorded. Transcripts were reviewed by investigators, the questionnaire was modified in response, and re-tested in a subsequent cycle of interviews until thematic saturation was achieved and no novel feedback was obtained.

**Population Health Research Capsule**

What do we already know about this issue?

Emergency department (ED) patient populations have a high prevalence of social risk yet optimal strategies for identifying these factors remain to be described.

What was the research question?

To develop and optimize a brief and understandable social risk and social need screening tool for ED patients.

What was the major finding of the study?

Patient informed changes supported a more concise, understandable, and dependable screening tool.

How does this improve population health?

This screening tool serves to assess social risk and social need in ED patients, enable linkage to relevant resources and improve overall health outcomes.

Patients were purposively sampled by language spoken (English or Spanish) as well as health literacy level (adequate or limited) in order to reduce bias in representation in the patient population and recruited from a large, urban ED. A bilingual research assistant screened patients for eligibility. Eligibility criteria included adult patients or parent/guardians of pediatric patients, fluency in either English or Spanish, provider approval for approach, and plans for discharge home. We excluded patients on an involuntary mental health hold or with active intoxication.

Patient participants completed a brief demographic survey and a health literacy assessment (Newest Vital Sign)^10^ in either English or Spanish, as well as the cognitive interview. Cognitive interviewing used the “think aloud” and “verbal probing” methods and was employed to understand the participant’s thought process, while going through the survey and comprehension of each survey question. The interviewer received cognitive interview training, and direct feedback following each interview, from a researcher trained in the technique. All interviews were recorded and professionally transcribed. All “think aloud” and “verbal probing” responses were reviewed and a cycle was complete when no new responses were given. All changes to the survey were made by consensus of the study team. The study was approved by the Institutional Review Board (IRB) of Partners HealthCare.

## RESULTS

In total, 16 patients completed cognitive interviews over the course of three cycles. Of the 16 participants, four (25%) were primarily Spanish-speaking and five (31%) were categorized as having limited health literacy (Online Supplement Table 2). Based on participant feedback, the survey was reduced and simplified from 16 questions, concerning the five domains of social risk and social need to 10 questions concerning four of the five original domains; neighborhood safety was excluded (Table).

Questions concerning neighborhood safety were removed as participants did not have consensus on the meaning of “safety.” For example, some participants felt these questions were referencing crime in the surrounding area [“You can safely walk around your neighborhood without feeling endangered” (adequate health literacy)]. Others felt they were referring to domestic violence [“Que se refiere como que si alguien que vive con un hijo, me lo golpeara, me va a hacer un maltrato agresivo como ¿violencia doméstica?”] [*That it refers to like if someone that lives with a son/daughter, were to hit him/her, [or] is going to aggressively mistreat me like domestic violence?* (adequate health literacy)] and [“I would assume, there, that you are referring to something that would be more like domestic abuse” (adequate health literacy)]. The lack of consistency in definition caused difficulty in interpreting a positive answer, and determining the appropriate community resource for response. The alternative option of adding further questions to clarify the type of safety need would have made the survey excessively long for ED use and overlapped with existing ED screening protocols for intimate partner violence. For these reasons, in addition to the limited community resources available to address safety, we removed the domain of safety from the question set.

For other domains, participants mentioned confusion in the wording and subsequent description of response options [“I think the wording is a little confusing after you’ve just gone through questions that are more direct yes or no…And so I had to switch gears and be like, ‘Oh wait. Okay. So now it’s often true, sometimes true, never true thing…’ (Adequate health literacy)]. Responses were thus simplified to binary options for improved participant understanding and ease in taking the survey [“Again, I just don’t like those sometimes, nevers, often, always. I think people get thrown off with that.” (Adequate health literacy)]. Questions were also removed for similarity to one another [“3a, I guess it’s fine. 3B is fine as well. They’re both pretty similar” (Adequate health literacy)].

## LIMITATIONS

Limitations of the study include recruitment limited to those who spoke English or Spanish. In this study, English- and Spanish-speaking patients had similar survey feedback; thus, all changes were made to both versions of the survey. The tool will need to be translated and tested in other languages. Interviews with participants with limited health literacy tended to be shorter with less feedback provided, suggesting that additional techniques to improve cognitive interviewing may be needed in this population. Social risk and social need screening results were not collected from the participants, so we cannot compare perceptions of the question to measured risk or need. We were only able to interview to thematic saturation across the categories of language and health literacy, which were chosen because they were believed to have the greatest impact on patient comprehension of the questions. Additionally, we do not have data on those who declined participation. Therefore, we were unable to compare those who did and did not participate in the study. Because there is no “criterion standard” for social risk and social need assessment,^11^ a larger study to understand the performance of the questions compared to other measurements of socioeconomic status, social risk, and social need will be the next step to better understand performance of this screening tool.

## CONCLUSION

The cognitive interviews provided important information concerning how to improve an assessment tool for measuring social risk and social need in the ED. After addressing a variety of the study participant concerns (including word choice, response categories, terminology, and question clarity), the final assessment tool (Online Supplement Final Survey) as compared to the original version, is more concise, understandable, and more likely to measure these factors as intended. Importantly, this tool includes both social risk and social need and was developed in both English and Spanish and among patients with a range of health literacy.

This short screening tool was developed to be of use to ED clinicians attempting to link patients to community resources, health system administrators developing programs to address adverse social determinants of health, and researchers working to improve care and outcomes for patients with social risk and social need. Given the importance and goal of integration of social determinant measures in clinical practice,^12^,^13^ we encourage future work to focus on testing the tool across multiple EDs, comparison with population level data, as well as implementation-science work regarding best practices for screening patients, and connecting them to appropriate community resources to improve health outcomes.

## Supplementary Information





## Figures and Tables

**Table:** Social risk and social need survey tool changes through three rounds of cognitive interview. (Abbreviated version of online supplement Table 3).

Original survey questions	Final survey questions
**Domain 1**	
1a. In the last month, have you slept outside, in a shelter or in a place not meant for sleeping?	[Removed]*
1b. In the last month, have you had concerns about the condition or quality of your housing?	*1a.* In the last month, have you had concerns about the condition or quality of your housing?
1c. In the last 12 months, how many times have you or your family moved from one home to another?	[Removed]**
1d. Are you worried that in the next 2 months, you may not have stable housing?	*1b.* Are you worried that in the *next month*, you may not have stable housing? ***
	*1c.* Would you like *resources to* help with housing?Δ
**Domain 2**	
2a. Within the past 12 months, you worried whether your food would run out before you got money to buy more.	*2a.* In the past 12 months, *have you* worried *that* your food would run out before you got money to buy more*?*ΔΔ
Response Options: Often true, sometimes true, never true, don’t know/refuse	Response Options: *“Yes, Often/Sometimes”* *“No, Never”* ΔΔΔ
2b. Within the past 12 months, the food you bought just didn’t’ last and you didn’t have money to get more.	*2b.* In the past 12 months, *has your food run out* and you didn’t have money to get more*?*ΔΔ
Response Options: Often true, sometimes true, never true, don’t know/refuse	Response Options: *“Yes, Often/Sometimes”* *“No, Never”* ΔΔΔ
	*2c.* Would you like *resources to* help with obtaining food?Δ
**Domain 3**	
3a. How often is it difficult to get transportation to or from your medical or follow-up appointments?	3a. How often is it difficult to get transportation to or from your medical or follow-up appointments?
Response Options: Does not apply, never, sometimes, often, always	Response Options: *“Always/often”* *“Sometimes/Never”* ΔΔΔ
3b. How often is it difficult to get transportation to or from your other non-medical activities (work, school etc)?	[Removed]
Response Options: Does not apply, never, sometimes, often, always	
	*3b.* Would you like *resources to* help with transportation?Δ
**Domain 4**	
4. In the past 12 months, have you had any utility (electric, gas, water or oil) shut off for not paying your bills?	*4a.* In the past 12 months, have you *worried that* any utility (electric, gas, water or oil) *would be* shut off for not paying your bills?†
	*4b.* Would you like *resources to* help with paying for your utility bills?Δ
**Domain 5**	
5a. Do you have any concerns about safety in your neighborhood?	[Removed]††
5b. Are you afraid you might be hurt in your apartment building or house?	[Removed]††
**Need**	
H1. Would you like help with shelter or housing?	[Moved]Δ
H.2 Would you like help with obtaining food?	[Moved]Δ
H.3 Would you like help with transportation?	[Moved]Δ
H.4 Would you like help paying for your utility bills?	[Moved]Δ
H.5 Would you like help regarding your personal or neighborhood safety?	[Moved]Δ then [Removed]††

-Note that changes to questions from the original to final survey are italicized in the final version.

* Respondents reported wanting a more definitive reference for a place “not meant for sleeping.”

** Participants reported people may be uncomfortable answering the question. Also other domain questions capture

homelessness sufficiently.

*** Number of months was changed from 2 to 1 to be consistent with previous questions.

Δ Questions reworded to clarify that interviewer is not providing said “help.” Also, reordered to directly follow questions about specific domain, for improved flow.

ΔΔ Reworded because of respondent confusion by question presentation.

ΔΔΔ Responses simplified to a binary option as respondents expressed difficulty with multiple options.

† Reworded as participants expressed experience “being close” to having a utility shut off.

†† The domain was removed, as there was a lack of consensus among participants about the meaning of safety.
