# Supplementary material for: Improving Understanding of Screening Questions for Social Risk and Social Need Among Emergency Department Patients
Source: West J Emerg Med. 2020 Aug 20;21(5):1170–4. doi: 10.5811/westjem.2020.5.46536 (PMC7514400; doi:10.5811/westjem.2020.5.46536)
Supplement: Supplementary file 1 [file wjem-21-1170-s001.docx]

**Online only supplement:**

Supplement Table 1: *Examples of prompts/probes asked about each question*

| **Think aloud** |
| --- |
| Interviewer addresses participant:  *We are going to go over the survey tool together using a ‘think aloud’ technique. If you could, please read these questions to yourself and share aloud what comes to mind and how you are thinking through and understanding each of one.* |
| **Verbal probing** |
| How do you understand [word/phrase]?  What do you think of when you hear [word/phrase]?  Is this question confusing to you or do you think it would be for others?  What about this question is confusing to you/others?  How can we make this question less confusing? |

*Supplement Table 2: Patient participant demographics*

|  | English | Spanish | Total N (%) |
| --- | --- | --- | --- |
| Age† | | | |
| 30-40 | 8 | 2 | 10 (67) |
| 41-50 | 3 | 1 | 4 (27) |
| 51+ | 0 | 1 | 1 (6) |
| Gender | | | |
| Male | 3 | 0 | 3 (19) |
| Female | 9 | 4 | 13 (81) |
| Other | 0 | 0 | 0 (0) |
| Race/Ethnicity* | | | |
| Hispanic | 1 | 4 | 5 (32) |
| Non-Hispanic White | 8 | 0 | 8 (50) |
| Non-Hispanic Black | 1 | 0 | 1 (6) |
| Non-Hispanic Asian | 1 | 0 | 1(6) |
| Non-Hispanic Other | 1 | 0 | 1 (6) |
| Insurance* | | | |
| Private | 8 | 0 | 8 (50) |
| Public/State | 4 | 4 | 8 (50) |
| Total | | | 16 (100) |

*0 participants identified as American Indian/Native Alaskan or Native Hawaiian of other Pacific Islander or, without medical insurance

†One participant preferred to not provide an age

|  | | | | | | | | | |
| --- | --- | --- | --- | --- | --- | --- | --- | --- | --- |
| *Supplement Table 3: Social risk and social need survey tool changes through each round of cognitive interviews* | | | | | | | | | |
| Original Question | **Round 1** | | | | **Round 2** | | | **Round 3** | **Final Survey** |
|  | Reason for Change | | Updated Question | | Reason for Change | Updated Question | | Reason for Change |  |
| DOMAIN 1 |  | |  |  |  |  |  |  |  |
| 1a. In the last month, have you slept outside, in a shelter or in a place not meant for sleeping? | Respondents reported wanting a more definitive reference for a place "not meant for sleeping".* | | 1a. In the last month, have you slept outside, in a shelter or in a car? | | Questions reordered for improved flow. | 1a. In the last month, have you had concerns about the condition or quality of your housing? | | [Unchanged] | **1a. In the last month, have you had concerns about the condition or quality of your housing?** |
| 1b. In the last month, have you had concerns about the condition or quality of your housing? | [Unchanged] | | 1b. In the last month, have you had concerns about the condition or quality of your housing? | |  | 1b. In the last month, have you slept outside, in a shelter or in a car? | | [Removed]  Participants reported people may be uncomfortable answering the question. Also previous questions capture homelessness sufficiently. ** |  |
| 1c. In the last 12 months, how many times have you or your family moved from one home to another? | [Removed]  Question not found to provide relevant information to the study. | |  | |  |  | |  |  |
| 1d. Are you worried that in the next 2 months, you may not have stable housing? | Question renumbered | | 1c. Are you worried that in the next 2 months, you may not have stable housing? | | Number of months was changed from 2 to 1 to be consistent with previous questions. | 1c. Are you worried that in the next month, you may not have stable housing? | | Question Renumbered. | **1b. Are you worried that in the next month, you may not have stable housing?** |
|  |  | | H1. Would you like resources to help with shelter or housing? | | "Shelter" was removed to reduce wordiness and to clarify the question. | H1. Would you like resources to help with housing? | | [Unchanged] | **1c. Would you like resources to help with housing?** |
| DOMAIN 2 | | | | | |  |  |  |  |
| 2a. Within the past 12 months, you worried whether your food would run out before you got money to buy more.  Response Options:  Often true, sometimes true, never true, don't know/refuse | Reworded because of respondent confusion by question presentation. Responses simplified as respondents expressed difficulty in with multiple options. | | 2a. Within the past 12 months, have you worried whether your food would run out before you got money to buy more?  Response Options:  “Yes, Often/Sometimes” v.  “No, Never” | | Questions were reworded to improve clarity and reduce wordiness.† | 2a. In the past 12 months, have you worried that your food would run out before you got money to buy more?  Response Options:  “Yes, Often/Sometimes” v.  “No, Never” | | [Unchanged] | **2a. In the past 12 months, have you worried that your food would run out before you got money to buy more?**  **Response Options:**  **“Yes, Often/Sometimes” v.**  **“No, Never”** |
| 2b. Within the past 12 months, the food you bought just didn’t’ last and you didn’t have money to get more.  Response Options:  Often true, sometimes true, never true, don't know/refuse |  |  | 2b. Within the past 12 months, has the food you bought run out and you didn’t have money to get more?  Response Options:  “Yes, Often/Sometimes” v.  “No, Never” | |  | 2b. In the past 12 months, has your food run out and you didn’t have money to get more?  Response Options:  “Yes, Often/Sometimes” v.  “No, Never” | | [Unchanged] | **2b. In the past 12 months, has your food run out and you didn’t have money to get more?**  **Response Options:**  **“Yes, Often/Sometimes” v.**  **“No, Never”** |
|  |  | | H.2 Would you like resources to help with obtaining food? | | [Unchanged] | H.2 Would you like resources to help with obtaining food? | | [Unchanged] | **2c. Would you like resources to help with obtaining food?** |
| DOMAIN 3 | |  |  |  |  |  |  |  |  |
| 3a. How often is it difficult to get transportation to or from your medical or follow-up appointments?  Response Options:  Does not apply, never, sometimes, often, always | Responses simplified as respondents expressed difficulty in with multiple options. | | 3a. How often is it difficult to get transportation to or from your medical or follow-up appointments?  Response Options:  Always/often,  Sometimes/Never | | [Unchanged] | 3a. How often is it difficult to get transportation to or from your medical or follow-up appointments?  Response Options:  Always/often,  Sometimes/Never | | [Unchanged] | **3a. How often is it difficult to get transportation to or from your medical or follow-up appointments?**  **Response Options:**  **Always/often,**  **Sometimes/Never** |
| 3b. How often is it difficult to get transportation to or from your other non-medical activities (work, school etc)?  Response Options:  Does not apply, never, sometimes, often, always |  |  | 3b. How often is it difficult to get transportation to or from your other non-medical activities (work, school etc.)?  Response Options:  Always/often,  Sometimes/Never | | [Unchanged] | 3b. How often is it difficult to get transportation to or from your other non-medical activities (work, school etc.)?  Response Options:  Always/often,  Sometimes/Never | | [Removed]  This was removed to further simplify and shorten the survey as was considered similar to question 3a. |  |
|  |  | | H.3 Would you like resources to help with transportation? | | [Unchanged] | H.3 Would you like resources to help with transportation? | | [Unchanged] | **3b. Would you like resources to help with transportation?** |
| DOMAIN 4 |  | |  |  |  |  |  |  |  |
| 4. In the past 12 months, have you had any utility (electric, gas, water or oil) shut off for not paying your bills? | Reworded as participants expressed experience ‘being close’ to having a utility shut off. | | 4. In the past 12 months, have you worried that any utility (electric, gas, water or oil) would be shut off for not paying your bills? | | [Unchanged] | 4. In the past 12 months, have you worried that any utility (electric, gas, water or oil) would be shut off for not paying your bills? | | [Unchanged] | **4a. In the past 12 months, have you worried that any utility (electric, gas, water or oil) would be shut off for not paying your bills?** |
|  |  | | H.4 Would you like resources to help with paying for your utility bills? | | [Unchanged] | H.4 Would you like resources to help with paying for your utility bills? | | [Unchanged] | **4b. Would you like resources to help with paying for your utility bills?** |
| DOMAIN 5 | | | | | |  | | | |
| 5a. Do you have any concerns about safety in your neighborhood? | [Unchanged] | | 5a. Do you have any concerns about safety in your neighborhood? | | [Removed] This domain was removed, as there was a lack of consensus among participants about the meaning of safety. |  |  |  |  |
| 5b. Are you afraid you might be hurt in your apartment building or house? | [Removed] Respondents reported various interpretations of safety. | | H.5 Would you like resources to help regarding the safety of your neighborhood? | |  |  |  |  |  |
| NEED |  | |  |  |  |  |  |  |  |
| H1. Would you like help with shelter or housing? | Questions reworded to clarify that interviewer is not providing said "help". Also, reordered to directly follow questions about specific domain, for improved flow. | | H1. Would you like resources to help with shelter or housing? | |  |  |  |  |  |
| H.2 Would you like help with obtaining food? |  |  | H.2 Would you like resources to help with obtaining food? | |  |  |  |  |  |
| H.3 Would you like help with transportation? |  |  | H.3 Would you like resources to help with transportation? | |  |  |  |  |  |
| H.4 Would you like help paying for your utility bills? |  |  | H.4 Would you like resources to help with paying for your utility bills? | |  |  |  |  |  |
| H.5 Would you like help regarding your personal or neighborhood safety? |  |  | H.5 Would you like resources to help regarding the safety of your neighborhood? | |  |  |  |  |  |

*Further specification for understanding was requested for some questions [“‘¿Dónde ha dormido? ¿Ha dormido en la calle, en un albergue?’. Estas preguntas, tienen sentido y es fácil de entender. Aunque ‘Un lugar que no sea para dormir’, es como la única como-- ¿Qué puede ser 'no para dormir'?’…”] [‘Where have you slept?’ Have you slept in the street, in a homeless shelter?’ These questions make sense and are easy to understand. Although, ‘a place not meant for sleeping’, it is like the only one that—What could be ‘not for sleeping?’… (Adequate health literacy)].

**The “place not meant for sleeping” was subsequently replaced with “a car” but the question was then removed for potential discomfort in responding [“And maybe they’d feel a little ashamed...and I don’t think someone would answer directly” (Adequate Health Literacy)] and the fact that the specificity would not capture all unstable housing circumstances.

†The phrase “whether your food” was changed to “that your food” for improved clarity [“I think that that second part over here, have you worried whether your food would run out before you got money to buy more? Maybe the actual structure can be changed a little bit” (Low Health Literacy)].

**Final Survey English**

1a. In the last month, have you had concerns about the condition or quality of your housing?

Yes No

1b. Are you worried that in the next month, you may not have stable housing?

Yes No

1c. Would you like resources to help with housing?

Yes No

2a. In the past 12 months, have you worried that your food would run out before you got money to buy more?

Yes, often/sometimes No, never

2b. In the past 12 months, has your food run out and you didn’t have money to get more?

Yes, often/sometimes No, never

2c. Would you like resources to help with obtaining food?

Yes No

3a. How often is it difficult to get transportation to or from your medical or follow-up appointments?

Sometimes/never Often/always

3b. Would you like resources to help with transportation?

Yes No

4a. In the past 12 months, have you worried that any utility (electric, gas, water or oil) would be shut off for not paying your bills?

Yes No

4b. Would you like resources to help with paying your utility bills?

**Final Survey Spanish**

1a. Durante el último mes, ¿le ha preocupado la condición o calidad en la cual se encuentra su hogar?

Sí No

1b. ¿Se preocupa Ud. que en el próximo mes usted no pueda tener un lugar en donde vivir?

Sí No

1c. ¿Le gustaría recibir recursos de ayuda con la vivienda?

Sí No

2a. ¿En los últimos 12 meses, le preocupaba que la comida se acabara antes de que tuviera dinero para comprar más?

Sí, muchas veces/ a veces No, nunca

2b. ¿En los últimos 12 meses, se acabó su comida y no tuvo el dinero para comprar más?

Sí, muchas veces/ a veces No, nunca

2c. ¿Le gustaría recibir recursos de ayuda en obtener alimentos?

Sí No

3a. ¿Qué tan seguido le cuesta a Ud. conseguir transporte para llegar a--o salir de--sus visitas médicas?

Casi siempre/ siempre A veces/ nunca

3b. ¿Le gustaría recibir recursos de ayuda con el transporte?

Sí No

4a. En los últimos 12 meses, ¿Le preocupaba que se desconectara algún servicio (gas, agua, luz) por no pagar a tiempo?

Sí No

4b. ¿Le gustaría recibir recursos de ayuda en pagar sus facturas de servicios públicos?

Sí No
