# Supplementary material for: Improving Understanding of Screening Questions for Social Risk and Social Need Among Emergency Department Patients
Source: West J Emerg Med. 2020 Aug 20;21(5):1170–4. doi: 10.5811/westjem.2020.5.46536 (PMC7514400; doi:10.5811/westjem.2020.5.46536)
Supplement: Supplementary file 2 [file wjem-21-1170-s002.docx]

**Methodological Supplement**

**COREQ supplemental information**

*Personal characteristics.* Interviews were conducted by Gia Ciccolo, MPH, (GC) a senior clinical research coordinator at Massachusetts General Hospital (MGH). She is female and bilingual (English/Spanish). Margaret Samuels-Kalow, MD MSHP, (MSK) has completed formal qualitative methods training as part of her MSHP degree and trained the interviewer (GC) in these qualitative techniques.

*Relationship with participants.* The interviewer did not have an established relationship with participants prior to conducting the interview. She was aware that the goal of the study was to improve a social survey based on patient input.

*Theoretical framework.* There was no formal theoretical framework for this study. Thematic saturation was utilized to identify when cognitive interview cycles were complete, when no new information was offered.

*Participant selection.* Participants were recruited face-to-face. The interviewer’s shifts in the ED varied by time-of-day to reduce bias, and patient participants were selected based on eligibility criteria and purposive sampling to balance across language and health literacy. Purposive sampling was utilized to ensure the representation of relevant and diverse patient perspectives.^1^ The 2 categories of language and health literacy were chosen for purposive sampling as they were decided to likely have the greatest impact on participant understanding of questions. We do not have data on how many people refused to participate. All participants were recruited from either the Pediatric or Fast Track (lowest acuity) section of an urban Emergency Department and approached after having seen the providers, typically while waiting for results or continued care.

*Setting.* The setting of the data collection was in the ED. There were no non-participants present. The demographics of the sample are in the Online Supplement.

*Data collection.*  The guide was initially designed by MSK and piloted by GC. There were no repeat interviews and audio recording was used to collect the data. The cognitive interviews were approximately 10 minutes long, utilizing the hybrid model^2^ outlined in the manuscript and with a similar qualitative methodology used to refine another patient reported measurement tool.^3^ Each cycle consisted of 2-4 shifts of data collection prior to data saturation was reached and the 3 cycles were complete within approximately 6 weeks of data collection. No transcripts were returned to participants.

*Data analysis.* The data was collected by GC. Both investigators, GC and MSK, analyzed transcripts for patient input concerning the survey and made changes to the tool accordingly as discussed in the manuscript.

*Reporting.* Quotations were presented and identified. The complete table of edits made to the piloted survey as well as the final version in both English and Spanish are included in the Online Supplement.

**REFERENCES**

1. Rhodes, K et. al. Patients Who Can’t Get an Appointment Go to the ER”: Access to Specialty Care for Publicly Insured Children. Ann Emerg Med. 2013 Apr;61(4):394-403.
2. Ryan K, Gannon-Slater N, Culbertson M. Improving Survey Methods With Cognitive Interviews in Small- and Medium-Scale Evaluations. American Journal of Evaluation 2012;33:414-30.
3. Samuels-Kalow ME, Rhodes KV, Henien M, et al. Development of a patient-centered outcome measure for emergency department asthma patients. Acad Emerg Med 2017;24:511-22.
